# Inheritable testicular metabolic memory of high-fat diet causes transgenerational sperm defects in mice

**DOI:** 10.1038/s41598-021-88981-3

**Published:** 2021-05-03

**Authors:** Luís Crisóstomo, Ivana Jarak, Luís P. Rato, João F. Raposo, Rachel L. Batterham, Pedro F. Oliveira, Marco G. Alves

**Affiliations:** 1 Department of Anatomy and Unit for Multidisciplinary Research in Biomedicine (UMIB), Institute of Biomedical Sciences Abel Salazar (ICBAS), University of Porto, Rua de Jorge Viterbo Ferreira 228, 4050-313 Porto, Portugal; 2 Department of Pharmaceutical Technology, Faculty of Pharmacy, University of Coimbra, Coimbra, Portugal; 3 Health School of the Polytechnic Institute of Guarda, Guarda, Portugal; 4 NOVA Medical School – New University Lisbon, Lisbon, Portugal; 5 APDP – Diabetes Portugal, Lisbon, Portugal; 6 UCL Centre for Obesity Research, Division of Medicine, University College London, London, UK; 7 National Institute of Health Research, UCLH Biomedical Research Centre, London, UK; 8 Department of Chemistry, QOPNA & LAQV, University of Aveiro, Aveiro, Portugal

**Keywords:** Metabolomics, Epigenetic memory

## Abstract

The consumption of energy-dense diets has contributed to an increase in the prevalence of obesity and its comorbidities worldwide. The adoption of unhealthy feeding habits often occurs at early age, prompting the early onset of metabolic disease with unknown consequences for reproductive function later in life. Recently, evidence has emerged regarding the intergenerational and transgenerational effects of high-fat diets (HFD) on sperm parameters and testicular metabolism. Hereby, we study the impact of high-fat feeding male mice (F_0_) on the testicular metabolome and function of their sons (F_1_) and grandsons (F_2_). Testicular content of metabolites related to insulin resistance, cell membrane remodeling, nutritional support and antioxidative stress (leucine, acetate, glycine, glutamine, inosine) were altered in sons and grandsons of mice fed with HFD, comparing to descendants of chow-fed mice. Sperm counts were lower in the grandsons of mice fed with HFD, even if transient. Sperm quality was correlated to testicular metabolite content in all generations. Principal Component Analysis of sperm parameters and testicular metabolites revealed an HFD-related phenotype, especially in the diet-challenged generation and their grandsons. Ancestral HFD, even if transient, causes transgenerational “inherited metabolic memory” in the testicular tissue, characterized by changes in testicular metabolome and function.

## Introduction

Modern societies promote a fast-paced daily life that often leads to poor lifestyle choices. Notably, readily available and palatable fast food contributes to a high-fat diet (HFD) that has led to a marked increase in the prevalence of overweight and obesity worldwide^1^. Obesity-related comorbidities have increased proportionally, including non-communicable diseases, such as type 2 diabetes (T2D)^2^. Of interest, male infertility has been also correlated to this increase in obesity prevalence. Several studies report a coincident temporal trend in sperm quality decline^3,4^. Others report how male obesity is associated with infertility^5,6^ and lower success of Assisted Reproduction Techniques (ART)^7,8^. Recently, evidence has emerged concerning the intergenerational and transgenerational effects of male obesity on the health of their progeny^9–11^, particularly on sexual health^12,13^. HFD cause acute changes in human sperm^14^, and permanent changes in murine testicular metabolite content^15^. The age of onset of obesity and its comorbidities are occurring at an ever younger age^2,16^. Therefore, it is of utmost relevance to understand the metabolic factors transmitted via paternal lineage to the progeny.

Rodent models have been widely applied in transgenerational studies involving ancestral exposure to HFD^17,18^, including studies restricted to paternal inheritance. Although the male gamete can only carry a minimal amount of small non-coding RNAs (sncRNAs) besides the DNA content^17^, these studies show metabolic phenotypic changes inherited by direct progeny (sons) and subsequent descendants (grandsons)^12,19–21^. Moreover, seminal fluid composition potentially affects early phases of embryo development^22^. Epigenetic mechanisms respond to metabolic inputs, such as metabolite availability at cellular level, which is affected by diet^22^. “Metabolic memory” has been coined with metabolic adaptations in somatic cells, in the context of glycemic control and T2D complications^23^. Previous studies demonstrate that both somatic and germline cells adapt in response to metabolic, environmental cues, particularly HFD, and these adaptations are potentially inherited by the offspring via the paternal lineage^12,19–21^. Therefore, we advocate that “metabolic memory” could be adapted to the context of transgenerational epigenetic inheritance. This “inherited metabolic memory” refers to the inheritance of acquired adaptations to environmental variables in ancestry, such as diet, by progeny which has not been exposed to the same stimuli, for several generations. In this study we adopted a multivariate analysis of untargeted ^1^H-NMR metabolomics, to assess the effects of lifelong or temporary paternal HFD upon testicular metabolome and sperm parameters of the direct offspring (sons) and second-generation offspring (grandsons) in mice. Biometric and glucose homeostasis data was also recorded individually across generations. Based on these data, we demonstrate “inherited metabolic memory” caused by ancestral exposure to HFD in the paternal lineage.

## Results

### HFD increases weight after weaning but does not promote weight gain across generations

**Figure 1:**
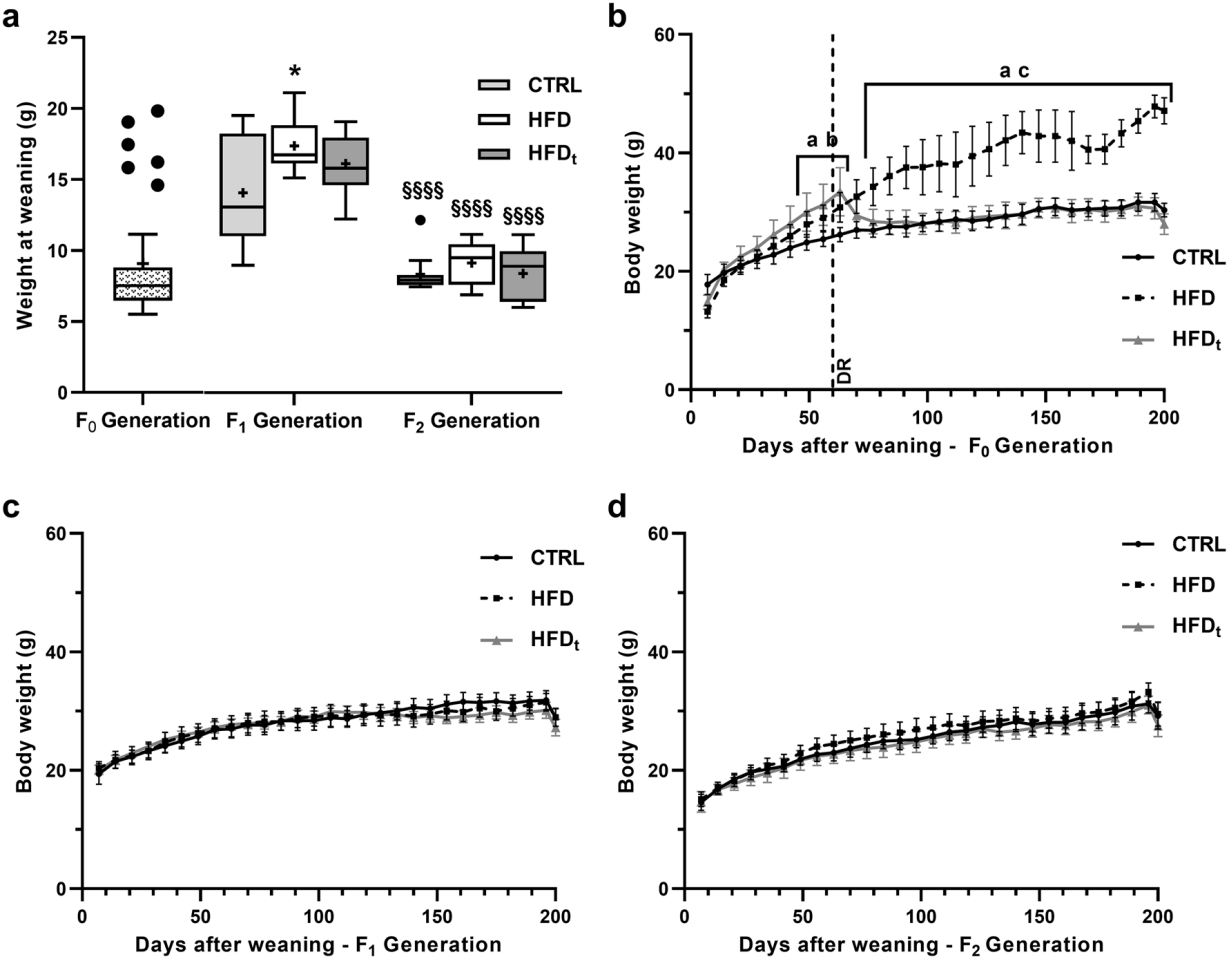
The evolution of body mass from weaning to sacrifice, across generations. (**a**) Body weight at weaning (g), expressed as Tukey’s whisker boxes (median, 25th to 75th percentiles ± 1.5 interquartile range). Mice of F_0_ Generation were weighted and then randomly assigned to an experimental group (n = 12 per group in each generation). Data was tested by one-way ANOVA with Tukey’s HSD for group comparison within the same generation. Two-way ANOVA with Šidak correction was used to compare each diet group in Generation F_1_ against its F_2_ counterparts. Significance was considered when p < 0.05. * vs. CTRL; ^#^ vs. HFD; ^§^ vs. Generation F_1_. Multiple significance signs represent different significance levels: *p < 0.05; **p < 0.01; ***p < 0.001; ****p < 0.0001; filled circle Extreme values; + Group mean. Body weight curves for (**b**) F0 Generation, (**c**) F_1_ Generation and (**d**) F_2_ Generation were obtained by monitoring the mice weight weekly (12 mice per group in each generation). Results are expressed as the mean body weight (g), with whiskers representing the 95% Confidence Interval. The main and simple effects between groups were tested by Repeated Measures ANOVA corrected for family-wise comparisons using the Šidak method, and by Greenhouse–Geisser correction for sphericity. Graphs were obtained using the software GraphPad Prism 8 (San Diego, CA, USA). Significance was considered when p < 0.05. a—CTRL vs. HFD; b—CTRL vs. HFD_t_; c—HFD vs. HFD_t_. *DR* diet reversion.

The experimental design of this study is detailed in “Materials and methods” section and in Supplemental Fig. S1. HFD mice sons (Generation F_1_) were significantly heavier than sons of CTRL mice (19 ± 2 g vs 15 ± 4 g). In generation F_2_, no differences were found between groups (Fig. 1a). Body weight curves for each group were obtained for F_0_ Generation (Fig. 1b), F_1_ Generation (Fig. 1c) and F_2_ Generation (Fig. 1d). As previously reported^15^, in F_0_ Generation both HFD and HFDt mice gain more weight than CTRL from early age. After diet reversion (DR), 60 days after weaning, HFDt mice start losing weight, and thereafter HFD mice became heavier than both CTRL and HFDt mice. In F_1_ Generation no differences were found between groups at any period, except at weaning (as previously mentioned). Similarly, no differences were found between the grandsons of the different groups (F_2_ Generation). The weight of various organs, fat mass and gonadosomatic index (GSI) were also assessed (Supplementary Table S1). In this dataset, more differences were found in F_1_ generation than in F2. HFD mice generated F_1_ mice with smaller left testis than CTRL (0.11 ± 0.02 g vs 0.12 ± 0.01 g). Comparing to mice from CTRL, mice from the HFD_t_ generated F_1_ mice with smaller testes (Left: 0.10 ± 0.02 g vs 0.13 ± 0.01 g; Right: 0.10 ± 0.01 g vs 0.12 ± 0.01 g), less epididymal (0.55 ± 0.14 g vs 0.76 ± 0.20 g) and perirenal (0.21 ± 0.07 g vs 0.29 ± 0.10 g) fat, lower fat mass (4.19 ± 0.96% vs 5.32 ± 1.01%) and GSI (0.74 ± 0.09% vs 0.85 ± 0.10%).

### The adoption of HFD does not affect glucose homeostasis in progeny

**Figure 2:**
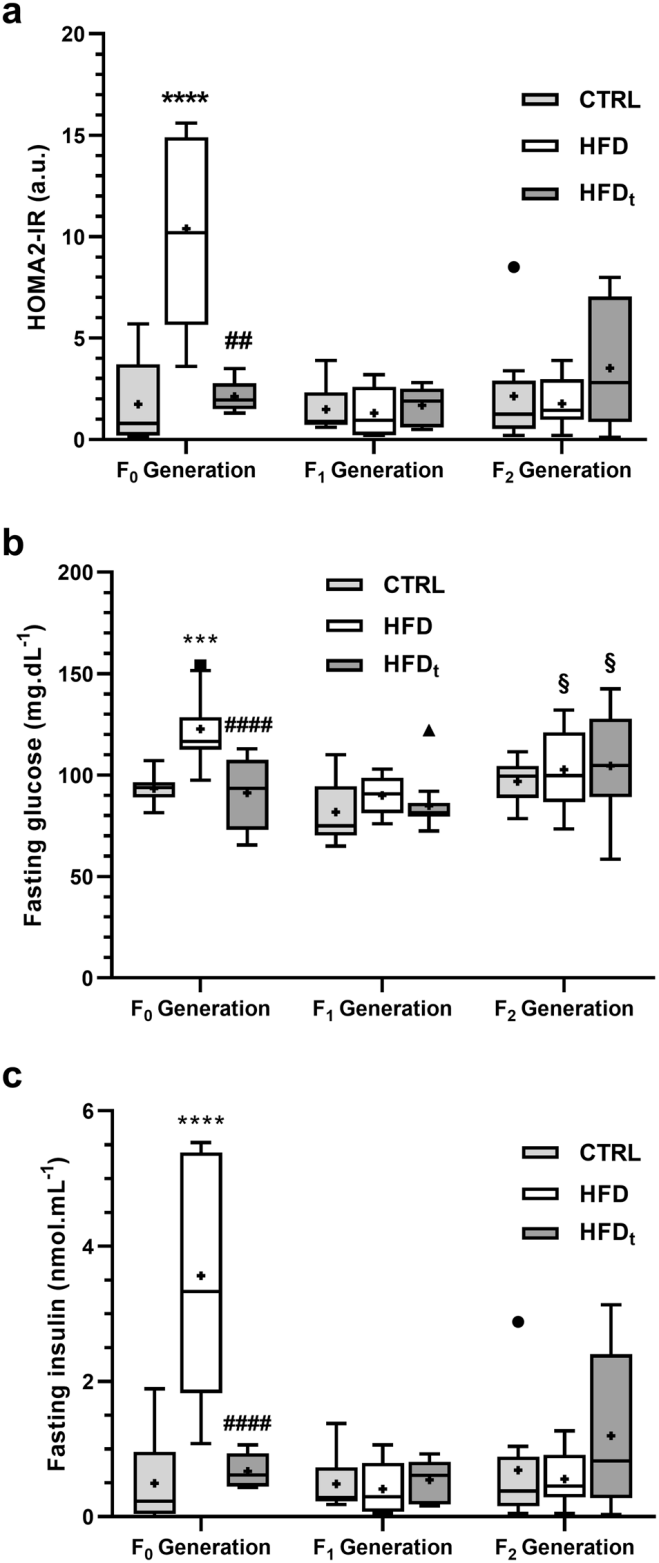
Glucose homeostasis across all the generations (n = 8 per group in each generation). (**a**) HOMA2-IR index; (**b**) fasting glycaemia; (**c**) serum insulin. Results are expressed as Tukey’s whisker boxes (median, 25th to 75th percentiles ± 1.5 interquartile range). Extreme values are represented individually according to the experimental group (filled circle CTRL—standard chow; filled square HFD—high-fat diet; filled triangle HFD_t_—transient high-fat diet). The samples were obtained in the day of sacrifice, 200 days post-weaning. Data was tested by one-way ANOVA with Tukey’s HSD for group comparison within the same generation. Two-way ANOVA with Šidak correction was used to compare each diet group in F_1_ Generation against its F_2_ counterparts. Graphs were obtained using the software GraphPad Prism 8 (San Diego, CA, USA). Significance was considered when p < 0.05. * vs. CTRL; ^#^ vs. HFD; ^§^ vs. F_1_ Generation. Multiple significance signs represent different significance levels: *p < 0.05; **p < 0.01; ***p < 0.001; ****p < 0.0001. + Group mean.

The adoption of lifelong HFD induced a pre-diabetic state in HFD mice (F_0_ Generation) characterized by increased insulin resistance (10.40 ± 4.64 u.a., Fig. 2a) and fasting glycemia (123 ± 16 mg/dL, Fig. 2b). Interestingly, an increase in average fasting glycemia was observed in groups CTRL and HFDt in generation F_2_, compared to their progenitors (97 ± 10 mg/dL and 105 ± 27 mg/dL respectively) (Fig. 2b). This difference is not observed in fasting insulinemia (Fig. 2c). No changes in this parameter were found in F_1_ and F_2_ Generations. Regarding the intraperitoneal Glucose Tolerance Test (ipGTT) and the intraperitoneal Insulin Tolerance Test (ipITT) (Supplementary Table S2), the adoption of a lifelong HFD led to glucose intolerance and insulin resistance, but diet reversion prevented this affect. Interestingly, during ipITT, the offspring of HFD, generation F_1_, reached higher serum glycemia levels after 90 and 120 min than the offspring of CTRL and HFD_t_.

### Paternal HFD during early life induces sperm defects that persist for 2 generations

**Figure 3:**
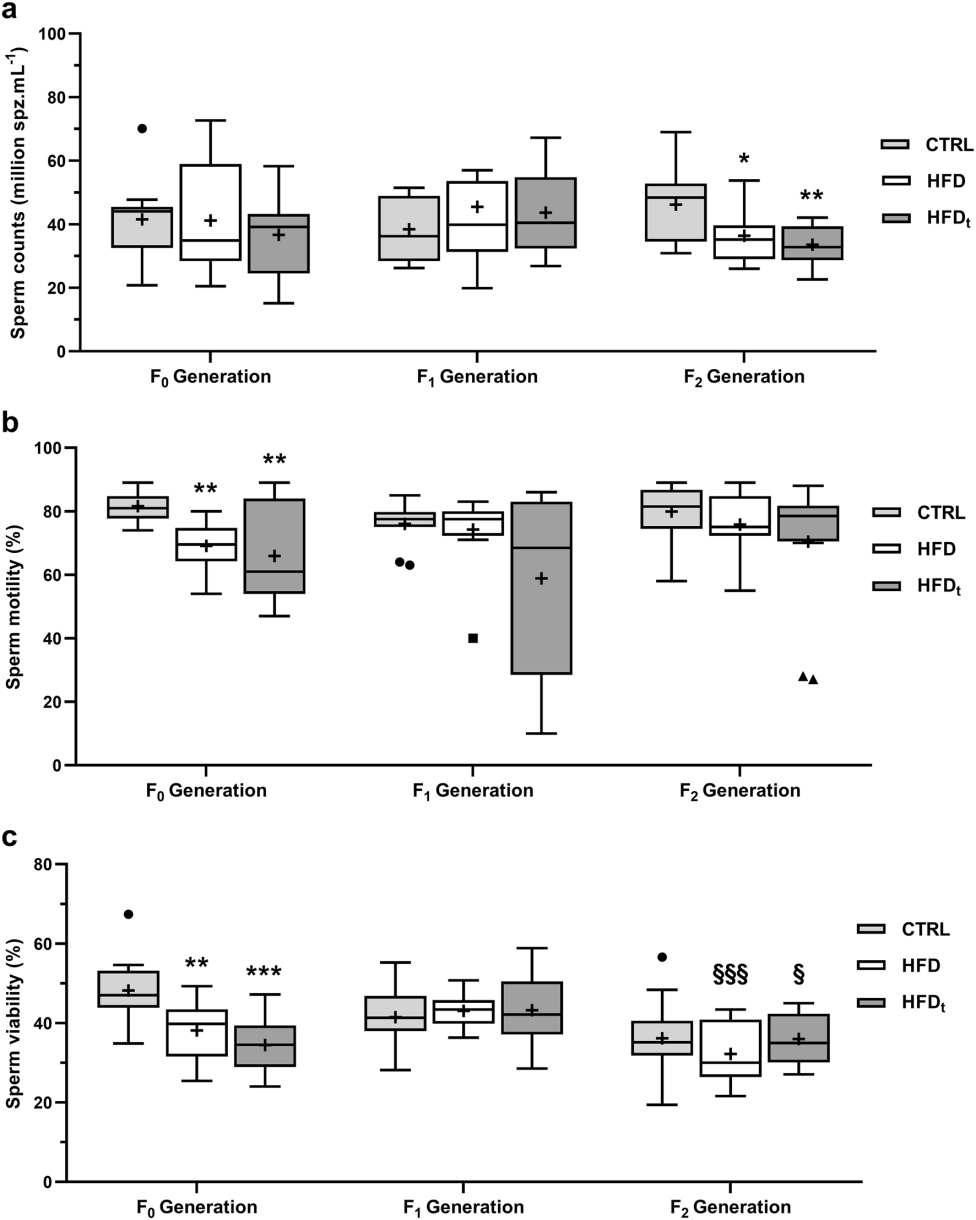
Sperm parameters across all the generations (n = 12 per group in each generation). (**a**) Sperm concentration; (**b**) sperm motility; (**c**) sperm viability. Results are expressed as Tukey’s whisker boxes (median, 25th to 75th percentiles ± 1.5 interquartile range). Extreme values are represented individually according to the experimental group (filled circle CTRL—standard chow; filled square HFD—high-fat diet; filled triangle HFD_t_—transient high-fat diet). Epidydimal sperm was obtained from mice immediately after sacrifice, at 200 days post-weaning. Data was tested by one-way ANOVA with Tukey’s HSD for group comparison within the same generation. Two-way ANOVA with Šidak correction was used to compare each diet group in F_1_ Generation against its F_2_ counterparts. Graphs were obtained using the software GraphPad Prism 8 (San Diego, CA, USA). Significance was considered when p < 0.05. * vs. CTRL; ^#^ vs. HFD; ^§^ vs. F_1_ Generation. Multiple significance signs represent different significance levels: *p < 0.05; **p < 0.01; ***p < 0.001; ****p < 0.0001. + Group mean.

Sperm counts, viability, motility and morphology were assessed across generations (Fig. 3, Table 1). In F_1_ generation, differences were only found in sperm morphology. The offspring of HFD_t_ mice had a greater proportion of normal sperm (37 ± 6%) and lower proportion of decapitated sperm (9 ± 2%) than those from HFD (32 ± 5% and 11 ± 6%) and CTRL (33 ± 5% and 13 ± 6%). The offspring of HFD mice also had a lower proportion of decapitated sperm (11 ± 6%) than CTRL (13 ± 6%), but higher proportions of pin head (9 ± 2%) and bent neck (8 ± 4%) defects. Interestingly, the F_2_ generation from HFD and HFD_t_ mice had decreased sperm counts compared to CTRL (36.42 ± 8.62 M/mL and 33.60 ± 6.12 M/mL respectively). Moreover, although no changes in sperm viability were found between groups in F_2_ generation, it is interesting to note that F_2_ from HFD and HFD_t_ mice had lower values of sperm viability (32 ± 8% and 36 ± 6% respectively) than their F_1_ progenitors (43 ± 4% and 32 ± 8%). Regarding sperm morphology in generation F_2_, those mice obtained from HFD mice had significantly lower proportion of normal sperm (34 ± 5%) than those F_2_ obtained from the other groups. Mice of the F_1_ generation had a significantly higher proportion of head defects (CTRL: 27 ± 6%; HFD: 27 ± 7%; HFD_t_: 25 ± 5%) than their offspring in F_2_ generation (CTRL: 18 ± 5%; HFD: 22 ± 4%; HFD_t_: 19 ± 3%). The proportion of head defects in the HFD mice, generation F_2_, explains the lower proportion of normal sperm in this group. Reproductive parameters (success rate, litter size and male ratio per litter) were assessed in Generations F_0_ and F_1_ (Table S3). However, no changes were found.

**Table 1:** Sperm morphology across generations (n = 12 per group in each generation).

	Sperm morphology	CTRL	HFD	HFD_t_	χ^2^ test
F_0_ Generation	Normal	40 ± 6	34 ± 7***	37 ± 4***	145.57****
	Decapitated	12 ± 5	11 ± 4	12 ± 3^#^	
	Pin head	3.7 ± 1.3	6.1 ± 1.3***	6.7 ± 1.9*** ^##^	
	Flattened head	3.9 ± 0.8	4.6 ± 1.2	6.0 ± 0.9*** ^###^	
	Bent neck	6.4 ± 1.0	9.3 ± 3.6***	5.3 ± 2.0^###^	
	Coiled tail	34 ± 6	35 ± 7	33 ± 2* ^##^	
	Normal	40 ± 6	34 ± 7***	37 ± 4***	94.02****
	Head defects	20 ± 4	22 ± 4	25 ± 2*** ^###^	
	Tail defects	40 ± 6	44 ± 6**	38 ± 3^###^	
	Normal	40 ± 6	34 ± 7***	37 ± 4***	31.22****
	Abnormal	60 ± 6	66 ± 7***	63 ± 4***	
F_1_ Generation	Normal	33 ± 5	32 ± 5	37 ± 6** ^###^	62.96****
	Decapitated	13 ± 6	10.84 ± 5.71*	9 ± 2*** ^#^	
	Pin head	7.7 ± 2.1	9.29 ± 2.40*	8.5 ± 2.1	
	Flattened head	6.7 ± 1.5	7.35 ± 1.89	7.2 ± 1.9	
	Bent neck	6.6 ± 1.6	8.37 ± 3.85**	7.3 ± 1.3	
	Coiled tail	33 ± 4	32.39 ± 5.17	31 ± 5	
	Normal	33 ± 5	32 ± 5	37 ± 6** ^###^	29.04 ****
	Head defects	27 ± 6	27 ± 7	25 ± 5* ^#^	
	Tail defects	40 ± 4	41 ± 5	38 ± 5	
	Normal	33 ± 5	32 ± 5	37 ± 6** ^###^	28.02****
	Abnormal	67 ± 5	68 ± 5	63 ± 6** ^###^	
F_2_ Generation	Normal	40 ± 8^§§^	34 ± 5***	38 ± 5^##^	76.180****
	Decapitated	6.2 ± 2.9^§§§^	7.5 ± 3.1	6.0 ± 2.6^# §^	
	Pin head	4.4 ± 1.4^§§§^	5.2 ± 1.1^§§§§^	6.3 ± 1.7*** ^# §^	
	Flattened head	7.4 ± 2.4	9.3 ± 2.18*	6.7 ± 2.9^###^	
	Bent neck	12 ± 4^§§§^	10 ± 4	13 ± 4^## §§§^	
	Coiled tail	30 ± 6	34 ± 3**	30 ± 4^##^	
	Normal	40 ± 8^§§^	34 ± 5***	38 ± 5^##^	31.62****
	Head defects	18 ± 5^§§§^	22 ± 4*** ^§^	19 ± 3^# §§^	
	Tail defects	42 ± 8	44 ± 4	43 ± 6^§^	
	Normal	40 ± 8^§§^	34 ± 5***	38 ± 5^##^	26.86****
	Abnormal	60 ± 8^§§^	66 ± 5***	62 ± 5^##^	

Data is presented as the mean (%) ± standard deviation. Epidydimal sperm was obtained from mice immediately after sacrifice, at 200 days post-weaning. The distribution of sperm defects across different experimental groups, in each generation, was tested by χ^2^ test of independence. Comparisons within sperm morphology categories were obtained with the Z-test for column proportions with Bonferroni correction. Two-way ANOVA with Šidak correction was used to compare each diet group in F_1_ Generation against its F_2_ counterparts.

Significance was considered when p < 0.05. * vs. CTRL; ^#^ vs. HFD; ^§^ vs. F_1_ Generation. Multiple significance signs represent different significance levels: *p < 0.05; **p < 0.01; ***p < 0.001; ****p < 0.0001.

### Changes in testicular metabolism caused by HFD during early life prevail for several generations

**Figure 4:**
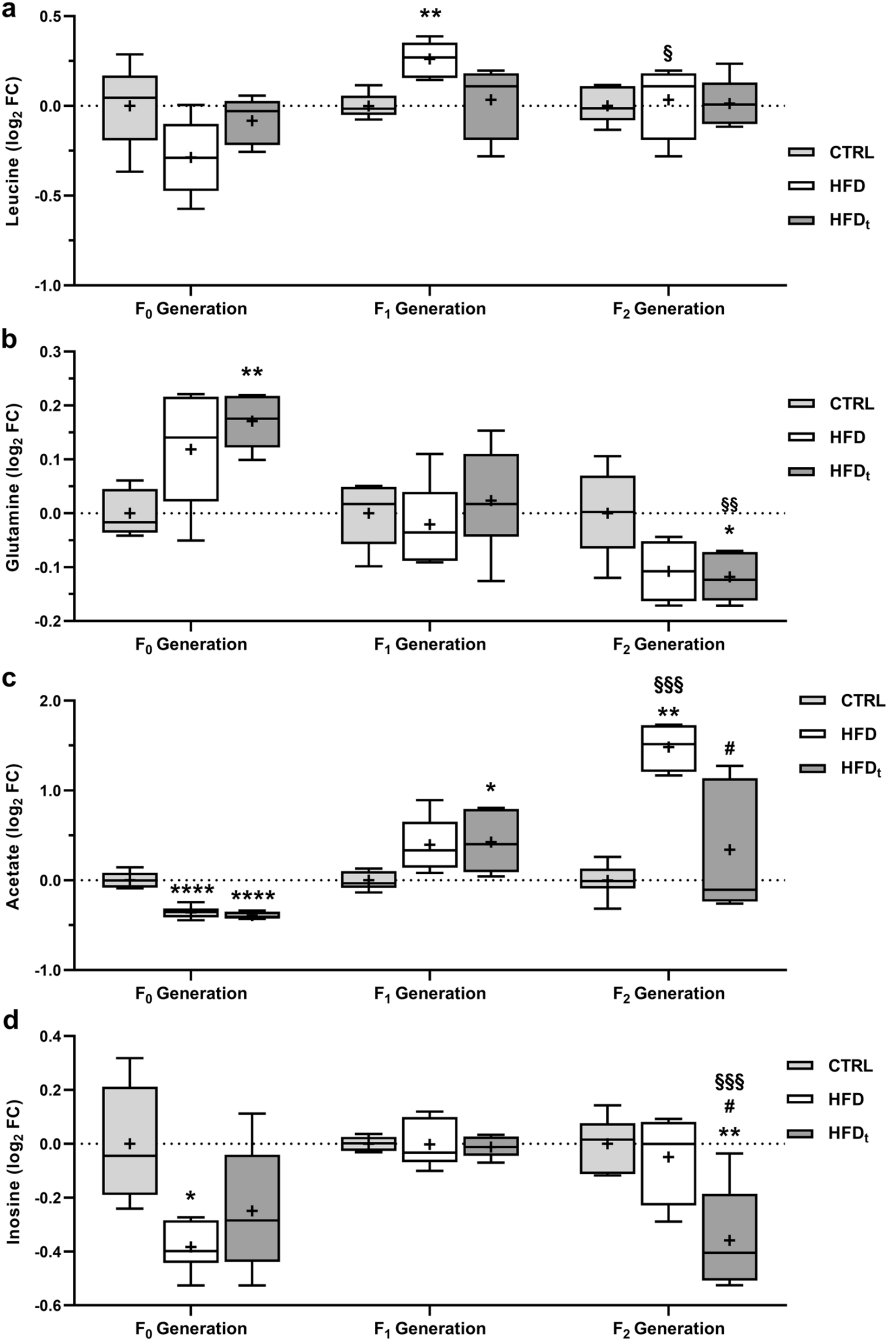
Testicular metabolites with variations across all generations (n = 6 per group in each generation). (**a**) Leucine; (**b**) Glutamine; (**c**) Acetate and (**d**) Inosine. Results are expressed as the log_2_ Fold Change (FC) to the CTRL of the corresponding generation, as Tukey’s whisker boxes (median, 25th to 75th percentiles ± 1.5 Interquartile range). Metabolites were extracted from the testes collected during sacrifice (200 days after mice weaning). Data was tested by univariate ANOVA with Tukey’s HSD for pairwise comparisons. Two-way ANOVA with Šidak correction was used to compare each diet group in F_1_ Generation against its F_2_ counterparts. Graphs were obtained using the software GraphPad Prism 8 (San Diego, CA, USA). Pairwise significance was considered when p < 0.05. * vs. CTRL; ^#^ vs. HFD; ^§^ vs. F_1_ Generation. Multiple significance signs represent different significance levels: *p < 0.05; **p < 0.01; ***p < 0.001; ****p < 0.0001. + Group mean.

Testicular polar metabolites were extracted and analyzed by ^1^H-NMR. Results are presented as the log_2_ Fold Change (FC) to CTRL of the corresponding generation. The full list of metabolites identified by ^1^H-NMR is available as Supplementary Table S4. Testicular leucine levels (Fig. 4a) were 20% higher in the sons of mice fed HFD (0.26 ± 0.10 log_2_ FC) than in sons of CTRL mice (F_1_ generation). The grandsons of mice fed HFD (F_2_ generation) had lower levels of testicular leucine (0.03 ± 0.21 log_2_ FC) than their F_1_ progenitors. Testicular glutamine levels (Fig. 4b), in F_0_ generation, were increased in the HFD_t_ group (0.23 ± 0.06 log_2_ FC). No changes were found in the sons of the diet-challenged mice (F_1_ generation). However, the grandsons of HFD_t_ mice had decreased levels of testicular glutamine, comparing to grandsons of CTRL (-0.18 ± 0.07 log_2_ FC). HFD-fed mice, even transiently, decreased testicular acetate content more than 1.5-fold, compared to CTRL, in generation F_0_ (Fig. 4c). The sons of both HFD and HFD_t_ (F_1_ generation) overcompensated their progenitors’ defect in testicular acetate, particularly the sons of HFD_t_ (0.48 ± 0.35 log_2_ FC). This effect persisted in the grandsons of HFD-fed mice (F_2_ generation), notably in the grandsons of HFD (1.33 ± 0.16 log_2_ FC), which had more than 2.5-fold more testicular acetate than the grandsons of CTRL. Despite the mean testicular acetate content of the grandsons of HFD_t_ being 1.2-fold increased comparing to CTRL, this difference was not significant due to the large standard deviation of the results (0.27 ± 0.78 log_2_ FC). Concerning inosine (Fig. 4d), lifelong HFD promoted the decrease in testicular levels (-0.68 ± 0.22 log_2_ FC) compared to CTRL (0.00 ± 0.31 log_2_ FC) in generation F_0_. Again, no differences were found for the testicular content of inosine in the sons of the diet-challenged mice (F_1_ generation). Interestingly, the grandsons of HFD_t_ mice had lower inosine levels in testes (− 0.68 ± 0.40 log_2_ FC) than the grandsons of CTRL (0.00 ± 0.15 log_2_ FC). Detailed values are shown in Table S5.

### Changes in testicular metabolism promoted by diet, even if transient, are correlated to sperm defects up to F_2_

**Figure 5:**
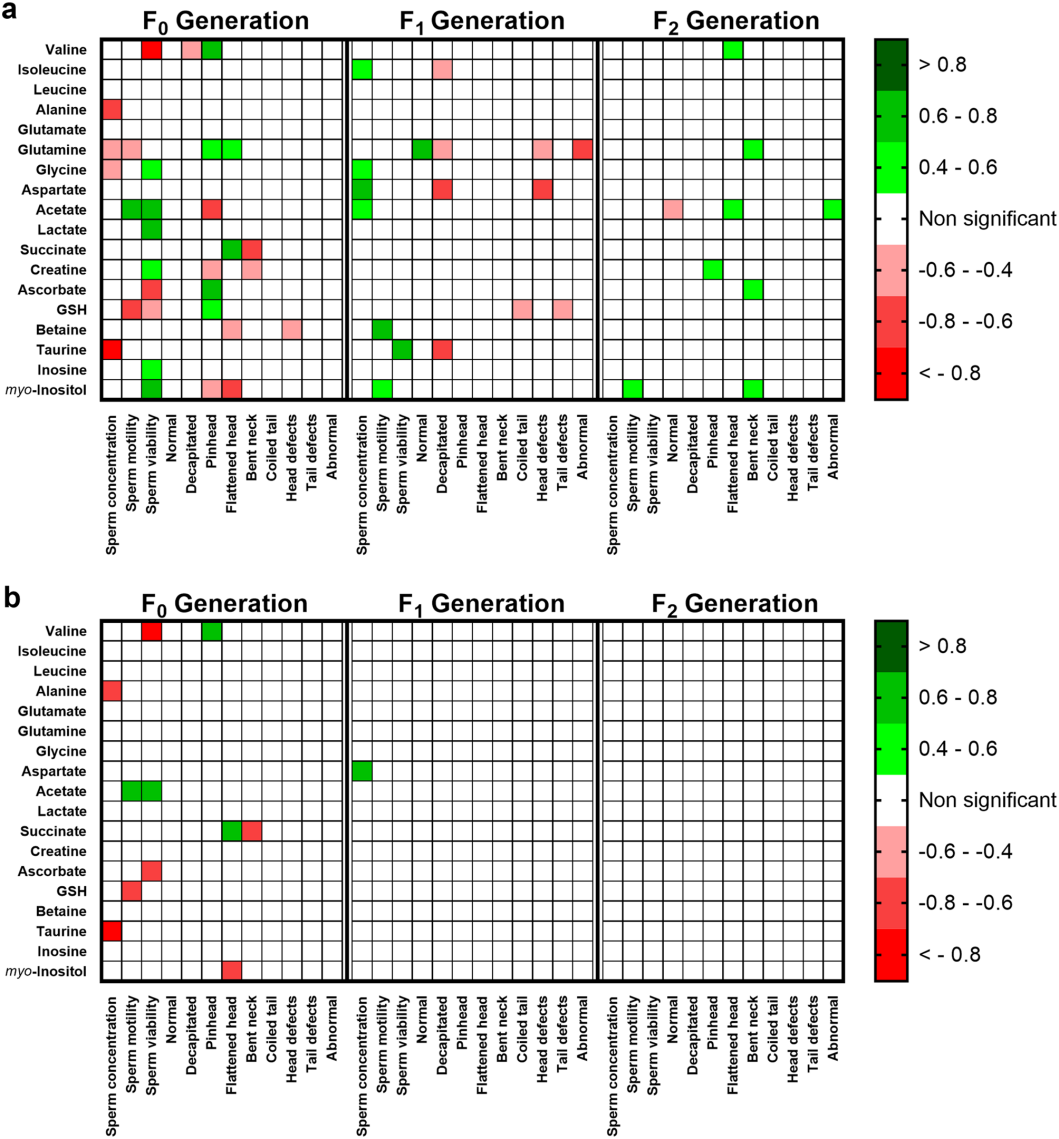
Correlations between sperm parameters and testicular metabolites, in each generation of the study (n = 6 per group in each generation). Correlation factors were obtained by Pearson’s method using SPSS 26 (Armonk, NY, USA). (**a**) Strong (|r|> 0.4) and significant correlations considering unadjusted two-tailed p < 0.05. (**b**) Strong (|r|> 0.4) and significant correlations considering FDR-adjusted p < 0.1. Heatmaps were obtained using the software GraphPad Prism 8 (San Diego, CA, USA).

Sperm parameters and testicular metabolites were correlated using Pearson’s method (Fig. 5). Correlation matrixes were obtained for each generation in the study independently, and relevant correlations were displayed when r <|0.4| and p < 0.05 (Fig. 5a), or when r <|0.4| and FDR p-adjusted < 0.1 (Fig. 5b). Whenever a correlation between variables is mentioned, it abides to the former mathematical criteria. The number of significant correlations decreased across generations, (F_0_: 31 correlations; F_1_: 17 correlations; F_2_: 9 correlations), especially when the p-value is adjusted for multiple testing (F_0_: 11 correlations; F_1_: 1 correlation; F_2_: 0 correlations). Before FDR p-adjustment, acetate, glutamine and *myo*-inositol showed significant correlations with sperm parameters in all generations, whereas glutamine had the highest number of significant correlations with sperm parameters (9), most of them in generation F_0_ (4). After FDR p-adjustment, acetate and succinate had the highest number of significant correlations with sperm parameters (2), all in F_0_ generation.

### Exposure to HFD, even if transient, induces intergenerational and transgenerational effects in testicular metabolism and sperm parameters

**Figure 6:**
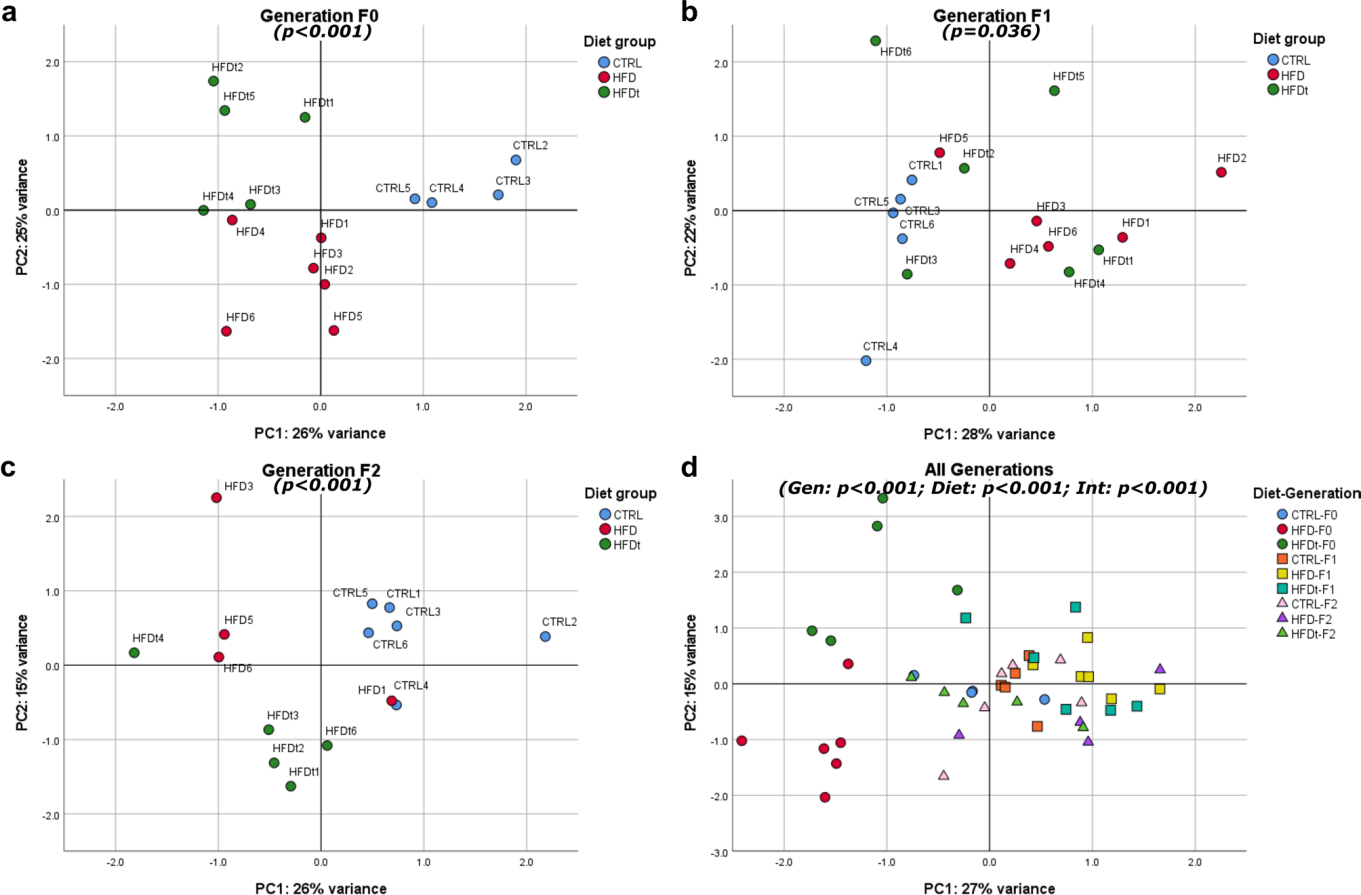
Sample dispersion in the two-dimensional space spanned by the two principal components extracted by PCA. Animals with at least one missing value in any of the variables considered (sperm parameters and testicular metabolites) were not included in the analysis. All the animals included in the model are represented in the graphs. Significance (p) values refer to overall permANOVA significance, obtained by the vegan R package. Significance was considered when adjusted p < 0.1. *PC1* principal component 1, *PC2* principal component 2, *Gen* generation factor, *Int* interaction (Generation*Diet) factor. PCA data visualization was obtained using the software SPSS 26 (Armonk, NY, USA) and edited using the software Inkscape 1.0 (Boston, MA, USA).

A multivariate analysis including sperm parameters (counts, viability, motility and normal morphology) and all tested testicular metabolites was performed using Principal Component Analysis (PCA). Two principal components were extracted to represent each sample in a two-dimensional Euclidean space (Fig. 6). A spatial dispersion graph was obtained for each generation individually, and as a transgenerational model, to evaluate clustering. permANOVA was then applied to detect overlapping of group clusters^24^ (Table S6).

In generation F_0_ clustering and well-separated groups are evident (F = 17.56, p < 0.001) (Fig. 6a). In generation F_1_, although there is overlap between groups, especially among the sons of HFD and HFD_t_ (p.adj = 0.430), the discrimination between groups was still significant (F = 2.70, p.adj = 0.036) (Fig. 6b). The grandsons of diet-challenged mice (generation F_2_) were better separated than their F_1_ progenitors (F = 6.45, p < 0.001) (Fig. 6c). Both the grandsons of HFD and the grandsons of HFD_t_ did not overlap with the grandsons of CTRL (p = 0.046 and p = 0.006, respectively). There is also a significant difference between HFD and HFD_t_ in F_2_ Generation (p = 0.089). When analyzing all the groups at once (Fig. 6d) there was a clear separation between the animals which have been HFD-challenged (HFD and HFD_t_, generation F0). Also, regardless of the generation, all CTRL samples are clustered near the origin. In this model, all the factors are significant predictors, although generation is the most relevant predictor (Generation: F = 20.19, p < 0.001; Diet: F = 7.79, p < 0.001; Generation*Diet: F = 7.00, p < 0.001). It was not possible to calculate multiple comparisons in this scenario.

## Discussion

Metabolic diseases have reached pandemic proportions worldwide. Excess energy intake is considered the primary driver for the development of metabolic diseases. However, it is now clear that there are hereditable latent signals that trigger metabolic dysfunction. We used a multivariate analysis approach based on untargeted testicular metabolomics and analyzed sperm quality in fathers exposed to different diets and their offspring up to F_2_. Our data show that there are paternally-mediated intergenerational and transgenerational effects on testicular metabolome and sperm quality caused by a HFD, even if transient (Table 2). Exposing fathers to HFD induces intergenerational and transgenerational effects. The former are defined as phenomena observed in offspring caused by direct exposure of the progenitor to an condition (i.e. environmental or contaminant)^25^ while the latter are caused by an ancestral exposure to which the previous generation has not been exposed^25,26^. Our study is based on paternal lineage and focuses on sons (intergenerational effects) and grandsons (transgenerational effects) of the diet challenged fathers.

**Table 2:** Intergenerational and transgenerational effects of HFDs in the paternal lineage.

Intergenerational effects	Transgenerational effects
↓ Left testis weight (HFD/HFDt)	↓ Sperm counts (HFD/HFDt)
↓ Right testis weight (HFD/HFDt)	↓ Normal sperm (HFD)
↓ Epidydimal fat weight (HFDt)	↑ Head defects (HFD)
↓ Perirenal fat weight (HFDt)	↑ testicular acetate (HFD)
↓ Fat mass (HFDt)	↓ testicular glutamine (HFDt)
↓ GSI (HFDt)	↓ testicular inosine (HFDt)
↑ Body weight at weaning (HFD)	↑ testicular ascorbate (HFDt)
↑ Normal sperm (HFDt)	
↓ Head defects (HFDt)	
↑ testicular glycine (HFD/HFDt)	
↑ testicular leucine (HFD)	
↑ testicular acetate (HFDt)	

The affected lineage is reported in brackets, comparing to the CTRL group of the corresponding generation.

Maternal metabolic dysfunction has been associated with a reprogramming that promotes diabetes or obesity in the offspring^27,28^ and the metabolic health of the father has been overlooked. However, an increasing number of studies show that the metabolic profile of the father at conception also influences the metabolic profile of the offspring. For instance, paternal obesity has an impact on the offspring metabolic health^9,29,30^ as fathers with obesity are more likely to have children with obesity^31^. Interestingly, we have found limited evidence on this phenomenon. No differences were found in HOMA2-IR, fasting glucose and fasting insulinemia in the sons and grandsons of mice fed with HFD. Yet, the sons of HFD mice had increased body weight at weaning than the sons of CTRL and HFD_t_, whereas the sons of HFD_t_ had lower fat mass than the other groups, which elicits an effect of the father’s diet in the body composition of the progeny. Also, the sons of HFD mice had increased serum glucose levels at 90 and 120 min during insulin tolerance test than the sons of CTRL and HFD_t_ mice, reflecting an abnormal insulin response. These intergenerational effects of HFD in body adiposity and glucose homeostasis have been associated with altered DNA methylation patterns^32^. Particularly, it has been shown that paternal exposure to HFD is associated with aberrant DNA methylation of adipogenic genes and genes related to increased diabetic risk^33,34^, even in humans^35^. We have found that sons of mice fed with HFD_t_ had lower GSI than sons of CTRL and HFD mice, suggesting potential fertility problems. Infertility has been described as one of the most striking silent co-morbidities caused by metabolic diseases^36^. We have previously showed that consumption of HFD causes irreversible changes in testicular metabolism even after switching to normal diet, using the same experimental model used here for the progenitors^15^. Sperm quality is the first line assessment of male fertility potential and a central indicator of testicular function. Our data show a transgenerational effect on sperm quality, particularly on sperm counts. The grandsons of individuals fed with HFD, even if transient, had a decreased sperm counts when compared with grandsons of individuals fed with a normal diet. A recent systematic review and meta-regression analysis showed that sperm counts are decreasing worldwide to dangerous levels^3^. Concomitantly, metabolic diseases incidence and prevalence are also rising^37–41^. Thus, a relationship between these two factors has been proposed and discussed^42^. Our data show that HFD, even if transient, has a transgenerational effect on sperm counts. We also detected inter- and transgenerational effects of diet on sperm morphology, particularly on head defects. Thus, our data suggest that unhealthy dietary habits induce inter- and transgenerational effects on sperm quality.

To further determine how diet-mediated inter- and transgenerational effects on sperm are mediated in the testis, we performed an exploratory analysis based on untargeted metabolomics from testicular extracts and multivariate analysis. Testicular metabolome reflected inter- and transgenerational effects of HFD consumption even when this occurred only until early adulthood. We found that the sons of mice fed with HFD, even temporarily, have higher levels of testicular glycine than sons of mice fed with standard chow. Glycine supplementation has been reported to improve sperm parameters in hypercholesterolemic rats^43^. Decreased glycine levels have been associated with decreased cell permeability, mitochondrial membrane potential and the expression of blood-testis-barrier factors in the TM4 cell line^44^, which derives from murine Sertoli Cells. The increase in testicular glycine content could be due to an overcompensation mechanism to overcome the damage caused by HFD in the ancestors. Indeed, despite the increased body weight at weaning of the sons of HFD mice, there were no differences in sperm parameters between mice in this generation (F_1_), and glycine positively correlated with sperm concentration. The sons of mice fed with HFD until early adulthood displayed changes in the testicular content of leucine, a branched-chain amino acid, compared to sons of CTRL. Branched-chain amino acids have been implicated in the onset of metabolic syndrome and insulin resistance^45^. Increased serum branched-chain amino acid levels are associated with obesity and diabetes in humans, in response to insulin resistance^46,47^. There are several testis-specific or abundant leucine-rich proteins^48,49^. Though the role for these proteins is not well characterized, accumulation of leucine may impact upon the assembly and function of these testicular leucine-rich proteins. Testicular acetate levels were increased in the sons of HFD_t_ mice and in the grandsons of HFD mice, compared to CTRL mice of the corresponding generation. Acetate is exported by Sertoli Cells to developing germ cells, where it is used as a membrane lipid precursor^50^. It was previously described that leptin promotes the excretion of acetate in isolated human Sertoli Cells^51^. Considering the reduced sperm counts of the grandsons of mice fed HFD, compared to the grandsons of CTRL, our data suggests that the turnover of germ cells is increased in these mice. Testicular glutamine levels were decreased in the grandsons of mice temporarily exposed to HFD. Glutamine is a crucial energy substrate for Sertoli Cells, whose metabolism is similar to the Warburg effect described in cancer cells^52^. Grandsons of HFD_t_ mice also had a decrease in testicular inosine, compared to grandsons of CTRL and grandsons of HFD. Inosine is a precursor of purines. In rat testis, high-energy diets decrease inosine levels, with concomitant increase in hypoxanthine, which promotes a pro-inflammatory environment^53^, due to increased xanthine oxidase activity. Indeed, testicular ascorbate levels are decreased in the grandsons of HFD mice, compared to the grandsons of HFD_t_ mice. Therefore, the decrease in glutamine may impair Sertoli Cell ability to provide nutritional support to developing germ cells. The inhibition of the rate-limiting reaction converting inosine to guanylyl metabolites has been used to arrest meiosis in mice oocytes^54^. Thus, lower testicular inosine availability may inhibit the differentiation of germ cells, notably by inhibiting meiosis. This is reflected by our data that shows that the grandsons of HFD_t_ mice had lower sperm counts than the grandsons of CTRL. However, the association between transgenerational epigenetic and metabolomic fingerprints is scarce in the literature. Yet, a study in rats has demonstrated that early life exposure to di-n-butyl phthalate, an endocrine disruptor, caused an increase in testicular betaine levels for three generations, with concomitant decrease in sperm counts and DNA hypomethylation^55^. Although the ancestral exposure in this study was an indirect in utero exposure (embryonic exposure), it illustrates that metabolic fingerprints are likely the result of epigenetic factors that persist in the paternal lineage for several generations.

PCA was used in conjugation with permANOVA to evaluate the discriminant power of testicular metabolite content and sperm parameters as a function of diet and the male ancestor’s diet (Fig. 6, Table S6). Indeed, this approach showed that animals fed with HFD, even transiently, share testicular metabolite signatures and sperm parameters, significantly different from animals fed a standard diet. Notably, sons of HFD_t_ mice display an intermediate phenotype between the sons of CTRL and the sons of HFD mice. However, the grandsons of HFD and HFD_t_ present specific testicular metabolome traits compared to CTRL grandsons, which are related to their poorer sperm parameters. Taken together, these results suggest that HFD, even transiently, causes a significant impact on sperm parameters and testicular metabolism in the diet-challenged generation (F_0_), but also has intergenerational (F_1_) and transgenerational (F_2_) effects on the offspring. Our findings corroborate previous studies implicating deleterious effects caused by ancestral HFD on sperm parameters in rodents^56,57^. Our data also support the theory that testicular metabolome is involved in this phenomenon, though the mechanism underlying the inheritance of this phenotype is still unknown. The epigenetic signatures carried by spermatozoa is one of the most plausible mechanisms involved in this inheritance. They have been recently associated to the inheritance of the acquired phenotypes, notably in the male lineage and in response to ancestral exposure to HFD^58^. The DNA methylation pattern is one of the mechanisms previously implicated in the epigenetic transmission of traits. Previous studies have demonstrated that dietary and environmental exposures can alter the extent and the *loci* of methylation of the DNA carried by spermatozoa, thus conditioning gene expression as early as during embryo development^59,60^. Yet, the extent to which DNA imprinting due to methylation can significantly affect development and health outcomes in the offspring is still debatable^18,61^. Nevertheless, the influence of sperm DNA methylation and sperm sncRNA content in the inheritance of testicular metabolomic traits should be considered, and it must be a focus of further studies.

Here we show that sperm parameters are correlated with testicular metabolome, which corroborates our earlier findings^15^. Hence, spermatozoa may carry signatures sensitive to HFD that encode the “metabolic memory” of testicular cells in the offspring, which drive the inter- and transgenerational phenotypes of sperm parameters. “Metabolic memory” was coined with the metabolic adaptations occurring at the cellular level in progressive stages of metabolic syndrome, and as a response to hyperglycemic states^23^. This term is, however, too narrow in the context of intergenerational and transgenerational inheritance. “Inherited Metabolic Memory” refers to the inheritance of acquired metabolic signatures in response to environmental variables by the progeny which has not been exposed to the original stimuli. In summary, we suggest that an ancestral exposure to HFD, even if transiently, causes “inherited metabolic memory” in testicular tissue of the offspring up to two generation. This “inherited metabolic memory” is likely transmitted by epigenetic markers carried by the sperm, and is characterized by changes in the testicular metabolome, testicular function, and sperm parameters of the non-exposed progeny. Further research must focus on the epigenetic mechanisms underlying the “inherited metabolic memory”.

## Materials and methods

### Animal Model

This study was performed in three generations of *Mus musculus* C57BL6/J mice (Supplementary Fig. S1). The first generation (Generation F_0_) was reared as previously described^15,62^. Mice were originated from normoponderal progenitors (both male and female), fed with a standard chow (#F4031, BioServ, USA—Carbohydrate: 61.6%, Protein: 20.5%, Fat: 7.2–16.3% Kcals) and water ad libitum. After weaning (21–23 days), F_0_ mice (n = 36) were randomly divided in three groups: control (CTRL) (n = 12), HFD (n = 12) and HFD_t_ (n = 12). Mice from the CTRL group were fed with a standard chow. Mice from the HFD group received a HFD (#F3282, BioServ, USA—Carbohydrate: 35.7%, Protein: 20.5%, Fat: 36.0–59.0% Kcals). The mice from HFD_t_ group were fed with a HFD for 60 days (#F3282, BioServ, New Jersey, USA) and then switched to standard chow (#F4031, BioServ, New Jersey, USA). F_0_ mice were mated starting at 120 days of age with normoponderal, chow-fed, randomly selected females of same age to generate the F_1_ generation. Mating lasted for 8 days and involved placing a male and a female in the same cage for 6 h each day, without water or food supply. After weaning, F_1_ mice were assigned to the same experimental group as their fathers: CTRL_F1—offspring of CTRL (n = 12); HFD_F1—offspring of HFD (n = 12); HFD_t__F1—offspring of HFD_t_ (n = 12). In this generation (F_1_), all mice were fed with standard chow. Food and water were supplied without restrictions. The mating of F_1_ mice was performed under the same conditions as their progenitors (Generation F_0_). Mice from the resulting generation F_2_ were assigned to experimental groups in the same way as their fathers (12 animals per group). All mice of the generation F_2_ were fed with standard chow after weaning. Food and water were supplied without restrictions. All mice in all generations were kept in 12 h dark:light cycles, 4 mice per cage. Mice from all generations were euthanized by cervical dislocation 200 days after weaning, and tissues were collected for further analysis. Total body weight, water and food intake were monitored weekly from weaning to sacrifice. The animal model is compliant with the ARRIVE guidelines, was internally reviewed by the Organization for Animal Welfare (ORBEA) and approved and licensed by the Portuguese Veterinarian and Food Department (DGAV) with the registration number 0421/000/000/2016. All animal experiments were performed according to the “Guide for the Care and Use of Laboratory Animals” published by the US National Institutes of Health (NIH Publication No. 85–23, revised 1996) and European rules for the care and handling of laboratory animals (Directive 86/609/EEC).

### Glucose homeostasis assessment

Glucose tolerance and insulin resistance were assessed experimentally using the intraperitoneal glucose tolerance test (ipGTT) and intraperitoneal insulin tolerance test (ipITT) protocols^63^, at 196 and 198 days post-weaning age. ipGTT was performed after overnight fast, whereas ipITT was performed in the morning after a 4 h fast. Fasting glycemia was measured before sacrifice (200 days post-weaning) using a glucometer (One Touch Ultra Lifescan-Johnson, Milpitas, CA, USA) by collecting a drop of blood from the tail vein. Blood was then collected by cardiac puncture and centrifuged at 1500×*g*, 4 ºC, for 10 min to collect the serum. Insulin was quantified in serum using a Rat/Mouse Insulin ELISA assay (EZRMI-13K, Millipore). Glucose homeostasis was evaluated according to the HOMA2 scores^64^, using the HOMA2 calculator (University of Oxford, United Kingdom).

### Evaluation of epididymal sperm parameters

Epididymides were isolated immediately after sacrifice (200 days post-weaning age) and placed in pre-warmed (37 °C) Hank’s Balanced Salt Solution (HBSS) pH 7.4, minced with a scalpel blade and the suspension was incubated for 5 min (37 °C). Sperm parameters were evaluated as previously described^15,65^. Sperm motility was calculated as the average proportion of motile sperm in 10 random microscope fields, observing a drop of sperm suspension on a warmed slide (37 °C) using an optical microscope (× 100 magnification). Epididymal sperm concentration was determined using a Neubauer counting chamber and an optical microscope (× 400 magnification), diluting the sperm suspension 1:50 in HBSS. Sperm viability and morphology were assessed in differently stained epididymal sperm smears, counting 333 spermatozoa in random fields using an optical microscope (× 400 magnification). Sperm viability smears were stained with eosin‐nigrosin^66^, as membrane-compromised spermatozoa stain pink. Sperm morphology smears were stained with Diff‐Quick (Baxter Dale Diagnostics AG, Dubinger, Switzerland). Sperm morphology categories were mutually exclusive, i.e., spermatozoa displaying more than one defect were assigned according to the most serious defect category (decapitated > pinhead > flattened head > bent neck > coiled tail)^66^.

### Evaluation of fertility outcomes

After the mating of animals from F_0_ and F_1_ generations, we assessed the number of pregnant females to calculate the fertility rate of mice in different groups. Matings were performed until a sufficient number of pups to continue the experiment was achieved (12 pups per group in each generation). The total number of matings performed in each group and generation is available in the Supplementary Table S3. The litter size and the male/female ratio was assessed after weaning off the pups, not to stress the female progenitor. Only completed pregnancies resulting in at least one surviving pup were considered successful.

### NMR spectroscopy

A combined extraction of polar and nonpolar metabolites from testicular tissue was performed as previously described^67^. The aqueous phase containing polar water-soluble metabolites was lyophilized and analyzed by NMR spectroscopy, following a semi-quantitative methodology as described previously^68^. Metabolites were identified by comparing recorded spectra with reference spectra and the Human Metabolome Database^69^ and according to Metabolomics Standards Initiative guidelines for metabolite identification^70^. Identification levels are indicated in the Supplementary Table S4. ^1^H spectra were processed as described previously and well-resolved metabolite multiplets were integrated ^68^. Obtained areas were normalized to total spectral area, and then represented as the log_2_ fold variation (FC) to the CTRL group of the corresponding generation. Univariate and multivariate statistical analysis was conducted based on these values (Supplementary Table S5).

### Statistical analysis

Data normality and homoscedasticity were tested using Kolmogorov-Smirnoff test with Lilliefors’s correction and Levene’s test. Parametric statistics were adopted if one of the above assumptions was met. Pairwise comparisons were tested using univariate ANOVA corrected by Tukey’s HSD. Testicular metabolites were further corrected for multiple hypothesis testing, controlling the False Discovery Rate (FDR), using Benjamini–Hochberg method^71^. Two-way ANOVA with Šidak correction was used to test simple main effects of diet across generations F1 and F2. Repeated-measures ANOVA with Šidak correction was used to compare groups in time-course assays (body weight curves, ipGTT and ipITT). Tabular data was tested for independence by χ^2^ test, and column proportions were tested using z-test with Bonferroni’s correction. Correlations between variables coding for sperm parameters and testicular metabolites were calculated using Pearson’s method. Correlation strength was classified according to ranks^72^, and adjusted p-values for FDR were calculated using Benjamini–Hochberg method. PCA based on testicular metabolites and sperm parameters was performed to assess sample clustering according to diet and generation. Sample adequacy was assessed by the Kaiser–Meyer–Olkin Test and by the Bartlett’s Test of Sphericity. Forced factor extraction was performed to extract two principal components, using Varimax with Keiser’s Normalization as Rotation Method. These methods were performed using IBM SPSS Statistics v26 (Armonk, NY, USA). Euclidean distances between samples were obtained from the rotated PCA solution. Groups were compared according to distances in the Euclidean space using permANOVA^24^. Pairwise comparisons were corrected by controlling the FDR using the Benjamini–Hochberg method. These methods were performed on R 4.0.1^73^, running the R-packages vegan 2.5-6^74^ and RVAideMemoire, 0.9-77^75^. The significance level for pairwise corrections (Bonferroni, Tukey’s HSD and Šidak) was set to p < 0.05. The significance level for FDR-controlling methods was considered when adjusted p < 0.1.

## Supplementary Information

Supplementary Information.

## Data Availability

All the data, biological samples and other material used in this work is available upon reasonable request.
