# Supplementary material for: Inheritable testicular metabolic memory of high-fat diet causes transgenerational sperm defects in mice
Source: Sci Rep. 2021 May 3;11:9444. doi: 10.1038/s41598-021-88981-3 (PMC8093209; doi:10.1038/s41598-021-88981-3)
Supplement: Supplementary file 1 — Supplementary Information. [file 41598_2021_88981_MOESM1_ESM.pdf]

Inheritable testicular metabolic memory of high-fat diet causes transgenerational sperm defects in mice

Luís Crisóstomo [1], Ivana Jarak [2], Luís P. Rato [3], João F. Raposo [4,5], Rachel L. Batterham [6], Pedro F. Oliveira [7], Marco G. Alves [1] ✉

[1] Department of Anatomy and Unit for Multidisciplinary Research in Biomedicine (UMIB), Institute of Biomedical Sciences Abel Salazar (ICBAS), University of Porto, Portugal; [2]Department of Pharmaceutical Technology, Faculty of Pharmacy, University of Coimbra, Portugal; [3] Health School of the Polytechnic Institute of Guarda, Guarda, Portugal; [4] NOVA Medical School – New University Lisbon, Lisbon, Portugal; [5] APDP – Diabetes Portugal, Lisbon, Portugal; [6] Centre for Obesity Research, Rayne Institute; Centre for Weight Management and Metabolic Surgery & National Institute of Health Research, University College London, London, UK; [7] QOPNA & LAQV, Department of Chemistry, University of Aveiro, Portugal

Supplementary Information

Figure S1: Schematic representation of the experimental setting of the animal model. After weaning, mice generated from normoponderal males and females were weighed and randomly assigned to an experimental group: CTRL – standard diet; HFD – high-fat diet; HFD_t – transient high-fat diet (F_0 Generation) 60 days after weaning, HFDt mice diet was reverted from high-fat diet to standard diet. 120 days after weaning, mice from all the groups were assigned to normoponderal females to generate the next generation (F_1 Generation). Mice were euthanized 200 days after weaning. Mice from the F_1 Generation were exclusively fed with standard diet. Similarly, they were mated with normoponderal females 120 days after weaning to generate the F_2 Generation, and were euthanized 200 days post-weaning. Mice from the F_2 Generation were fed with standard diet and euthanized at 200 days post-weaning.

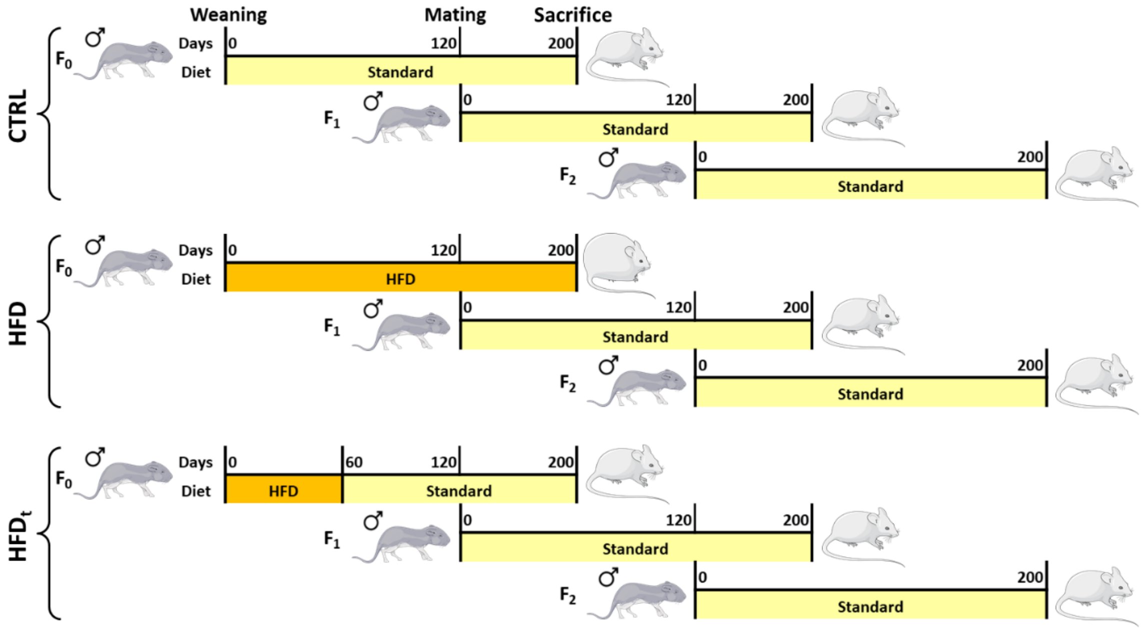

Table S1: Biometric parameters across generations (n = 12 per group in each generation). Results are represented as the mean (g) ± standard deviation. Data was tested with one-way ANOVA with Tukey's Honest Significant Differences. Significance was considered when $p < 0.05$. * vs. CTRL; # vs. HFD. * p<0.05; ** p<0.01; *** p<0.001; **** p<0.0001.

	Group	F_0 Generation	F_1 Generation	F_2 Generation
Body mass (g, at sacrifice)	**CTRL**	30.34 ± 1.80	28.92 ± 2.36	29.21 ± 3.58
	**HFD**	**47.10 ± 3.48 ******	28.93 ± 2.42	29.50 ± 3.19
	**HFD_t**	**27.99 ± 2.59 ####**	27.22 ± 2.20	27.51 ± 2.90
Left testis (g)	**CTRL**	0.11 ± 0.01	0.13 ± 0.01	0.13 ± 0.02
	**HFD**	0.12 ± 0.01	**0.11 ± 0.02 ***	0.12 ± 0.01
	**HFD_t**	0.11 ± 0.01	**0.10 ± 0.02 *****	0.12 ± 0.01
Right testis (g)	**CTRL**	0.11 ± 0.01	0.12 ± 0.01	0.13 ± 0.01
	**HFD**	0.11 ± 0.01	0.11 ± 0.01	0.13 ± 0.03
	**HFD_t**	0.11 ± 0.01	**0.10 ± 0.01 ***	0.11 ± 0.01
Right epididymis (g)	**CTRL**	0.06 ± 0.01	0.06 ± 0.02	0.07 ± 0.02
	**HFD**	0.06 ± 0.01	0.05 ± 0.02	0.07 ± 0.01
	**HFD_t**	0.06 ± 0.01	0.05 ± 0.01	0.07 ± 0.02
Liver (g)	**CTRL**	1.15 ± 0.08	1.16 ± 0.17	1.16 ± 0.19
	**HFD**	**1.38 ± 0.29***	1.13 ± 0.14	1.19 ± 0.17
	**HFD_t**	**1.08 ± 0.09 ###**	1.03 ± 0.16	1.11 ± 0.13
Seminal vesicles (g)	**CTRL**	0.25 ± 0.05	0.28 ± 0.05	0.29 ± 0.08
	**HFD**	0.31 ± 0.04	0.31 ± 0.06	0.31 ± 0.04
	**HFD_t**	0.27 ± 0.09	0.27 ± 0.06	0.31 ± 0.03
Epididymal fat (g)	**CTRL**	0.73 ± 0.23	0.76 ± 0.20	1.00 ± 0.43
	**HFD**	**2.21 ± 0.79 ******	0.73 ± 0.24	1.09 ± 0.44
	**HFD_t**	**0.63 ± 0.29 ####**	**0.55 ± 0.14 ***	0.82 ± 0.36
Perirenal fat (g)	**CTRL**	0.29 ± 0.14	0.29 ± 0.10	0.33 ± 0.22
	**HFD**	**1.56 ± 0.37 ******	0.30 ± 0.11	0.38 ± 0.20
	**HFD_t**	**0.23 ± 0.13 ####**	**0.21 ± 0.07 #**	0.31 ± 0.20
Retroperitoneal fat (g)	**CTRL**	0.32 ± 0.12	0.38 ± 0.11	0.38 ± 0.18
	**HFD**	**2.52 ± 0.71 ******	0.36 ± 0.12	0.39 ± 0.21
	**HFD_t**	**0.35 ± 0.19 ####**	0.29 ± 0.09	0.29 ± 0.18
Brown adipose tissue (g)	**CTRL**	0.14 ± 0.02	0.12 ± 0.03	0.16 ± 0.04
	**HFD**	**0.18 ± 0.04 ****	0.12 ± 0.03	0.18 ± 0.06
	**HFD_t**	0.16 ± 0.04	0.11 ± 0.04	**0.12 ± 0.05 #**
Fat mass (%)	**CTRL**	4.83 ± 1.35	5.32 ± 1.01	6.22 ± 1.72
	**HFD**	**13.70 ± 1.51 ******	5.12 ± 1.14	6.82 ± 2.38
	**HFD_t**	**4.78 ± 1.83 ####**	**4.19 ± 0.96 ***	5.56 ± 2.11
Gonadosomatic index (%)	**CTRL**	0.75 ± 0.08	0.85 ± 0.10	0.90 ± 0.11
	**HFD**	**0.48 ± 0.05 ******	0.77 ± 0.09	0.86 ± 0.11
	**HFD_t**	**0.79 ± 0.06 ####**	**0.74 ± 0.09 ***	0.84 ± 0.10

Table S2: Serum glycemia during the intraperitoneal Glucose Tolerance Test (ipGTT) and during the intraperitoneal Insulin Resistance Test (ipITT) (n = 12 per group in each generation). Data is represented as the mean glycemia ($mg.dL^{-1}$) ± standard deviation. The tests were performed at 196- and 198-days post-weaning age. ipGTT was performed after overnight fast, whereas ipITT was performed after a 4-hour fast during the morning. Results were analysed by Repeated Measures ANOVA with Šidak correction for pairwise comparisons within timepoints, and Greenhouse-Geisser correction for sphericity. Significance was considered when p < 0.05. * vs. CTRL; # vs. HFD. * p < 0.05; ** p < 0.01; *** p < 0.001; **** p < 0.0001.

		ipGTT time (minutes)				
**Gen**	**Group**	**0**	**30**	**60**	**90**	**120**
F_0	***CTRL***	93 ± 10	325 ± 36	232 ± 30	167 ± 24	132 ± 15
	HFD	**128 ± 30***	**409 ± 32*****	**384 ± 80******	**267 ± 57******	**226 ± 38******
	HFDt	**97 ± 34#**	**317 ± 57####**	**249 ± 38####**	**171 ± 23####**	**141 ± 29####**
F_1	***CTRL***	82 ± 16	346 ± 54	293 ± 34	217 ± 36	158 ± 17
	HFD	87 ± 9	331 ± 65	275 ± 63	192 ± 40	161 ± 43
	HFDt	87 ± 13	363 ± 86	280 ± 72	196 ± 38	157 ± 23
F_2	***CTRL***	88 ± 7	345 ± 49	287 ± 29	216 ± 39	162 ± 13
	HFD	91 ± 11	338 ± 45	281 ± 52	196 ± 41	161 ± 45
	HFDt	93 ± 9	333 ± 25	273 ± 64	205 ± 45	150 ± 22

		ipITT time (minutes)				
**Gen**	**Group**	**0**	**30**	**60**	**90**	**120**
F_0	*CTRL*	129 ± 14	79 ± 11	81 ± 13	99 ± 21	114 ± 24
	HFD	**145 ± 9***	**106 ± 19****	**109 ± 15****	**132 ± 27****	**161 ± 35****
	HFDt	**121 ± 14###**	**79 ± 26##**	**77 ± 30##**	**99 ± 11##**	**119 ± 32##**
F_1	*CTRL*	137 ± 12	78 ± 12	54 ± 34	83 ± 41	119 ± 28
	HFD	144 ± 23	81 ± 8	83 ± 30	**127 ± 31****	**154 ± 27****
	HFDt	136 ± 14	81 ± 19	61 ± 15	**71 ± 16###**	**96 ± 14###**
F_2	*CTRL*	135 ± 21	80 ± 9	56 ± 7	84 ± 10	98 ± 6
	HFD	140 ± 20	83 ± 6	50 ± 9	87 ± 7	104 ± 9
	HFDt	137 ± 25	87 ± 8	53 ± 12	82 ± 7	98 ± 5

Table S3: Reproductive parameters. Matings were performed until a sufficient number of pups to continue the experiment was achieved (12 pups per group in each generation). The total number of matings performed per group in each generation is stated in the table. Results are expressed as the mean (%) ± standard error of the mean. Data was tested for independence using χ^2 test, whereas family-wise comparisons were performed by Z-test for column proportions with Bonferroni correction.

Generation	Parameter	CTRL	HFD	HFD_t	χ^2 test
F_0	*Matings*	23	18	18	-
	Success rate (%)	48 ± 10	56 ± 12	47 ± 12	0.32
	Litter size[a]	5.8 ± 2.0	4.9 ± 1.1	4.3 ± 1.4	-
	Male pups (%)	53 ± 9	48 ± 9	48 ± 11	0.22
F_1	*Matings*	11	12	12	-
	Success rate (%)	18 ± 12	25 ± 13	17 ± 11	0.29
	Litter size[a]	7.8 ± 1.3	8.2 ± 2.1	8.3 ± 1.2	-
	Male pups (%)	51 ± 8	58 ± 7	35 ± 8	4.31

[a]Tested by one-way ANOVA, Tukey's Honest Significant Differences, and expressed as the mean (liveborn pups) ± standard deviation.

Table S4: Resonance assignment in the ^{1}H NMR spectra of polar testicular extracts (s: singlet, d: doublet, dd: doublet of doublets, t: triplet, m: multiplet).

	**Compound**[a]	**δ ^{1}H ppm (multiplicity, assignment)**
**1**	acetate	1.92 (s, αCH_3)
**2**	alanine	1.48 (d, βCH_3), 3.78 (t, αCH)
**3**	ascorbate	4.52 (s, CH_4)
**4**	aspartate	2.66 (dd, βCH), 2.81 (dd, ′CH), 3.86 (m, αCH)
**5**	betaine	3.26 (s, CH_3), 3.90 (s, αCH_2)
**6**	creatine	3.03 (s, CH_3), 3.93 (s, CH_2)
**7**	glutamate	2.07 (m, βCH), 2.14 (m, β'CH), 2.35 (m, γCH_2), 3.76 (m, αCH)
**8**	glutamine	2.13 (m, βCH_2), 2.45 (m, γCH_2), 3.75 (m, αCH)
**9**	glutathione	2.16 (q, βCH_2 Gl), 2.56 (m, γCH_2 Glu), 2.95 (m, βCH_2 Cys), 3.79 (m, αCH Gly), 4.59 (m, αCH_2 Cys)
**10**	glycine	3.55 (s, CH_2)
**11**	isoleucine	0.94 (t, δCH_3), 1.01 (d, $\beta' CH_3$), 1.26 (m, γCH), 1.49 (m, γ'CH), 1.98 (m, βCH), 3.67 (m, αCH)
**12**	inosine	8.30 (s, CH)
**13**	lactate	1.33 (d, βCH_3), 4.11 (q, αCH)
**14**	leucine	0.96 (t, δCH_3), 1.73 (m, $\beta\gamma CH_2$), 3.73 (m, αCH)
**15**	*myo*-inositol	3.26 (t, C5H), 3.52 (dd, C1H, C3H), 3.62 (m, C4H, C6H), 4.06 (t, C2H)
**16**	succinate	2.40 (s, CH_2)
**17**	taurine	3.26 (t, NCH_2), 3.43 (t, SCH_2)
**18**	valine	0.99 (d, γCH_3), 1.04 (d, $\gamma' CH_3$), 2.26 (m, βCH), 3.62 (m, αCH)

[a] all metabolites are putatively annotated (level 2 of identification according to Chemical Analysis Working Group (CAWG) Metabolomics Standards Initiative recommendations)

Table S5: Testicular sperm metabolites across generations, quantified by semi-quantitative ^{1}H-NMR (n = 6 per group in each generation). Results are expressed as the mean (log_2 Fold Change) ± standard deviation. Results were analysed by Univariate ANOVA, corrected for FDR by Benjamini-Hochberg method, and with Tukey's HSD correction for pairwise comparisons. Two-way ANOVA with Šidak correction was used to compare each diet group in F_1 Generation against its F_2 counterparts. FDR cut-off was set to $p < 0.1$. [a] Generation F0; [b] Generation F1; [c] Generation F2. Family-wise significance was considered when $p < 0.05$. * vs. CTRL; # vs. HFD; § vs. F_1 Generation. * p<0.05; ** p<0.01; *** p<0.001; **** p<0.0001. Abbreviations: FC – Fold Change (to CTRL); SD – standard deviation.

	Generation F_0			Generation F_1			Generation F_2		
**Metabolite**	**CTRL**	**HFD**	**HFD_t**	**CTRL**	**HFD**	**HFD_t**	**CTRL**	**HFD**	**HFD_t**
Valine	0.00 ± 1.11	0.51 ± 0.18	0.64 ± 0.25	0.00 ± 0.13	**0.27 ± 0.17***	0.15 ± 0.18	0.00 ± 0.17	0.24 ± 0.30	0.21 ± 0.28
Isoleucine	0.00 ± 0.19	-0.56 ± 0.76	0.01 ± 0.11	0.00 ± 0.17	0.29 ± 0.20	0.20 ± 0.26	0.00 ± 0.17	0.14 ± 0.25	0.15 ± 0.26
Leucine	0.00 ± 0.24	-0.29 ± 0.22	-0.08 ± 0.13	0.00 ± 0.07	**0.26 ± 0.10****	0.13 ± 0.16	0.00 ± 0.10	**0.03 ± 0.21§**	0.01 ± 0.14
Alanine	0.00 ± 0.29	-0.14 ± 0.15	0.05 ± 0.19	0.00 ± 0.07	0.09 ± 0.15	0.02 ± 0.13	0.00 ± 0.27	-0.21 ± 0.20	-0.17 ± 0.08
Glutamate [a]	0.00 ± 0.06	-0.08 ± 0.09	**0.04 ± 0.06#**	0.00 ± 0.02	0.04 ± 0.03	0.01 ± 0.03	0.00 ± 0.09	0.02 ± 0.07	0.04 ± 0.06
Glutamine [a]	0.00 ± 0.06	0.16 ± 0.14	**0.23 ± 0.06****	0.00 ± 0.09	-0.03 ± 0.11	0.03 ± 0.14	0.00 ± 0.12	-0.16 ± 0.09	**-0.18 ± 0.07* §§**
Glycine [b]	0.00 ± 0.11	-0.03 ± 0.10	-0.02 ± 0.06	0.00 ± 0.03	**0.14 ± 0.06****	**0.10 ± 0.06***	0.00 ± 0.07	**-0.07 ± 0.04§§§§**	**-0.08 ± 0.06§§§§**
Aspartate	0.00 ± 0.49	-0.20 ± 0.22	-0.21 ± 0.21	0.00 ± 0.19	0.11 ± 0.22	0.08 ± 0.05	0.00 ± 0.19	0.08 ± 0.25	0.11 ± 0.07
Acetate [a c]	0.00 ± 0.13	**-0.64 ± 0.15********	**-0.71 ± 0.09********	0.00 ± 0.15	0.46 ± 0.31	**0.48 ± 0.35***	0.00 ± 0.29	**1.33 ± 0.16** §§**	**0.27 ± 0.78#**
Lactate	0.00 ± 0.16	-0.22 ± 0.17	-0.24 ± 0.23	0.00 ± 0.06	0.06 ± 0.12	0.01 ± 0.11	0.00 ± 0.16	0.15 ± 0.29	-0.13 ± 0.17
Succinate [a]	0.00 ± 0.36	-0.58 ± 0.59	**1.00 ± 0.92##**	0.00 ± 0.33	-0.48 ± 0.27	-0.19 ± 0.36	0.00 ± 0.22	-0.28 ± 0.31	**0.15 ± 0.22§**
Creatine [a]	0.00 ± 0.09	**-0.18 ± 0.03******	**-0.06 ± 0.04#**	0.00 ± 0.05	0.00 ± 0.02	0.01 ± 0.02	0.00 ± 0.02	0.00 ± 0.03	0.01 ± 0.03
Ascorbate [a]	0.00 ± 0.27	**0.45 ± 0.15****	**0.41 ± 0.18***	0.00 ± 0.12	-0.15 ± 0.18	-0.09 ± 0.39	0.00 ± 0.36	-0.30 ± 0.53	**0.42 ± 0.36§§**

Metabolite	Generation F_0			Generation F_1			Generation F_2		
	CTRL	HFD	HFD_t	CTRL	HFD	HFD_t	CTRL	HFD	HFD_t
Glutathione [a]	0.00 ± 0.79	**0.97 ± 0.27***	**0.97 ± 0.24***	0.00 ± 0.04	-0.38 ± 0.38	-0.53 ± 0.78	0.00 ± 0.34	-0.31 ± 0.30	-0.43 ± 0.55
Betaine [a]	0.00 ± 0.04	0.15 ± 0.12	**-0.08 ± 0.14**[#]	0.00 ± 0.02	0.03 ± 0.05	0.00 ± 0.08	0.00 ± 0.04	0.03 ± 0.04	0.00 ± 0.05
Taurine	0.00 ± 0.15	-0.04 ± 0.13	0.10 ± 0.09	0.00 ± 0.09	0.05 ± 0.03	0.09 ± 0.06	0.00 ± 0.03	-0.01 ± 0.06	**-0.05 ± 0.08**[§§§]
Inosine [a c]	0.00 ± 0.31	**-0.68 ± 0.22***	-0.44 ± 0.45	0.00 ± 0.04	-0.01 ± 0.13	-0.02 ± 0.06	0.00 ± 0.15	-0.09 ± 0.28	**-0.68 ± 0.40**** [# §§§]
**myo-*Inositol*** [a]	0.00 ± 0.24	0.08 ± 0.06	**-0.19 ± 0.10**[#]	0.00 ± 0.02	0.00 ± 0.05	-0.01 ± 0.07	0.00 ± 0.02	-0.05 ± 0.05	0.00 ± 0.04

Table S6: Comparison of groups according to the 2-factor PCA in the two-dimensional Euclidean space, using permANOVA (R package vegan). Multiple comparisons corrected for the FDR by the Benjamini-Hochberg method, using the R package RVAideMemoire. Groups were considered significantly different when p.adj < 0.1.

Generation	Factor	Df	SumOfSqs	R^2	F	P	Comp	P.adj
F_0	*Diet*	2	20.87	0.75	17.56	0.001	*CTRL vs. HFD*	0.006
	Residual	12	7.13	0.25			*CTRL vs.* HFD_t	0.006
	Total	14	28.00	1.00			*HFD vs.* HFD_t	0.006
F_1	*Diet*	2	8.91	0.28	2.70	0.036	*CTRL vs. HFD*	0.018
	Residual	14	23.09	0.72			*CTRL vs.* HFD_t	0.224
	Total	16	32.00	1.00			*HFD vs.* HFD_t	0.430
F_2	*Diet*	2	14.51	0.52	6.45	0.001	*CTRL vs. HFD*	0.046
	Residual	12	13.49	0.48			*CTRL vs.* HFD_t	0.006
	Total	14	28.00	1.00			*HFD vs.* HFD_t	0.089

	Factor	Df	SumOfSqs	R^2	F	P
Trans-generational	*Diet*	2	10.44	0.11	7.00	0.001
	Generation	2	30.07	0.33	20.19	0.001
	*Generation*Diet*	4	23.20	0.25	7.79	0.001
	Residual	38	28.29	0.30		
	Total	46	92.00	1.00		
